# Effect of Hemodiafiltration Versus Hemodialysis on Cognitive Function Among Patients With End-Stage Renal Disease: A Multicenter Study

**DOI:** 10.7759/cureus.19719

**Published:** 2021-11-18

**Authors:** Abdullah Kashgary, Ahlam Khojah, Boshra Bamalan, Saleha Alafari, Marah Sindi, Albandri Alahmari, Ibtisam Gasm, Lujain Alkhateeb, Yazeed Khojah, Mostafa Abdelsalam

**Affiliations:** 1 Nephrology, King Abdulaziz University, Jeddah, SAU; 2 Medicine, King Abdulaziz University, Jeddah, SAU; 3 Faculty of Medicine, King Abdulaziz University Hospital, Jeddah, SAU; 4 Department of Family Medicine, King Abdulaziz University, Jeddah, SAU; 5 Mansoura Nephrology and Dialysis Unit, Mansoura University, Mansoura, EGY

**Keywords:** iadl score, moca score, hdf, hemodialysis, cognitive functions

## Abstract

Background: Cognitive impairment (CI) and dementia are common in patients with end-stage renal disease (ESRD) undergoing hemodialysis. Their cause is multifactorial. Our study is first to compare the impact of hemodialysis (HD) and online hemodiafiltration (HDF) on patients’ cognitive outcomes.

Methods: This was a cross sectional, multicenter cohort study. Adult ESRD patients aged >18 years on regular high flux HD or online HDF were recruited in the study. Clinical, laboratory, daily activities and cognitive functions assessment were assessed in all the involved individuals.

Results: A total of 241 patients were successfully recruited into the study: 164 treated with high flux HD and 77 treated with HDF. Hypertension and diabetes were the commonest associated comorbidities. 85.9% of patients were functionally independent with no significant difference between those on HD versus HDF. 81.3% of our patients showed different degrees of CI. HDF has no superiority in the improvement of cognitive functions. Age, vitamin D level and haemoglobin (Hb) were the most independent predictors of cognitive function impairment among HD patients.

Conclusions: Cognitive function impairment is a common problem in hemodialysis and is associated with multiple risk factors. HDF showed no beneficial effect over HD. There is no superiority of online HDF versus high flux HD in improving cognitive functions.

## Introduction

The mental process of acquiring knowledge using reasoning, perception and underlies all daily activities is known as cognition [1]. Cognitive impairment (CI) and dementia are common findings in end-stage renal disease (ESRD) and hemodialysis patients [2]. Poor cognitive function in the dialysis population is not limited to a certain age and older adults but occurs across the age spectrum [3,4].

Cognitive function impairment negatively affects functional dependence and behavioral symptoms, leading to poor outcomes and decreased compliance with medications and medical care [5,6]. The prevalence of cognitive function impairment among ESRD patients varies from 16 to 38% [7]. Moderate to severe chronic CI was found in 70% of patients receiving hemodialysis (HD) aged >55 years [8]. Many tools have been used to assess cognitive functions in HD patients. The Mini-Mental State Examination (MMSE) is the most frequently used worldwide, but the Montreal Cognitive Assessment (MOCA) was better in detecting mild CI. On the other hand, MOCA easily allows follow-up in those patients who may not speak English. MOCA was recommended to be the screening test for HD patients [9,10].

Theoretically, CI among HD patients is a multifactorial disease [11]. Hemodialysis is associated with increased risk of recurrent cerebral ischemia [12], vertebrobasilar infarcts [13], white matter disease [14] and cerebral oedema associated with disequilibrium syndrome [15]. Additionally, cognitive dysfunction had been linked to oxidative stress, uremic toxins, aluminum toxicity and hormonal imbalance. In addition to the previous risk factors, hemodialysis patients share the same risk factors as the general population, including older age [16], dyslipidemia [17] and the APO4 allele [18].

Improving cognitive function in hemodialysis patients is still a mystery. The safety of cholinesterase inhibitors and methyl D-aspartate receptor antagonists in dialysis is unknown [10]. High doses of vitamin B, erythropoietin, daily nocturnal hemodialysis, group-based cognitive-behavioural intervention and kidney transplantation were the main lines of treatment of cognitive function impairment hemodialysis patients [19-22]. Hemodiafiltration (HDF) is one of the available options for treating ESRD patients and was first described in the 1970s [23]. Previous studies have shown that HDF has no effect on all-cause mortality with an uncertain effect on non-fatal associated comorbidities in HDF patients [24,25].

In comparison to HD, online HDF is characterized by better solute removal, particularly middle-molecules [26-28]. Also, HDF is associated with hemodynamic stability, which leads to better outcomes [29]. HDF may shorten dialysis recovery time (DRT) by reducing intradialytic hypotensive episodes and improving health-related quality of life compared to HD [30,31].

To the best of our knowledge, this is the first study comparing the cognitive function of ESRD patients depending on the mode of treatment: online HDF versus the regular HD. On the other hand, no studies were done in Saudi Arabia (SA) to estimate cognitive impairment prevalence among dialysis patients.

## Materials and methods

This was a cross sectional, multicenter cohort study conducted between January 2020 and October 2020 in three hospitals in the western region of Saudi Arabia, the city of Jeddah (King Abdulaziz University Hospital, East Jeddah Hospital, and Mahjar Hospital).

This study was approved by the General Administration of Research and Studies, Ministry of Health, Kingdom of Saudi Arabia (H-02-J-002). This article was prepared in accordance with the STROBE checklist [32].

Study inclusion and exclusion criteria

Inclusion Criteria

According to local policy, all patient started high-flux HD. Patients who did not achieve adequacy targets and adequate phosphorus control after three months were changed to post-dilution HDF using 18-23 L exchange volume per treatment.

We included adult ESRD patients aged >18 years on thrice a week, four-hour dialysis sessions either on regular high flux HD or online HDF for >six months who agreed and provided written, informed consent to be involved in the study.

Exclusion Criteria

1. Patients who refused to be involved in the study, 2. Patients with language barriers, 3. Patients with known history of established advanced dementia, 4. Non-communicable patients like visual impairment, deaf-mute, etc, 5. Patients with recent hospital admission in the previous four weeks or recent infection.

Patients were subdivided into two groups according to the dialysis modality: Group (1) which included those on regular high flux HD, Group (2) which included those on online-HDF.

Study procedures

History and Clinical Evaluation

Each subject was evaluated for clinical history and underwent a complete medical examination. Socio-demographic data including age, gender, nationality, BMI, drugs, educational level, duration of ESRD, and dialysis age were gathered directly from the patient. Comorbidities were obtained by the Modified Charlson Comorbidity Index directly from the patients or medical reports.

Laboratory Investigations

Patients were evaluated for Kt/V, phosphate (P), calcium (Ca), parathyroid hormone (PTH), haemoglobin (Hb), ferritin, transferrin saturation (TSAT) lipid profile, and liver function (LFT) based on the most recent measurement at the time of the study. Measurements of Kt/V, P, Ca, Hb, ferritin, TSAT, and LFT were performed monthly, while PTH measurements were generally made quarterly according to our local protocol.

Activities of Daily Living

All patients were assessed for independent living skills by the Lawton Instrumental Activities of Daily Living (IADL) Scale [33] and for daily living activities as measured by the Katz Index of ADLs [34]. IADL score ranges from 0 (low function, dependent) to 8 (high function, independent) for women and 0 through 5 for men, respectively [33]. Katz score ranges from 0 (low function, dependent) to 6 (high (patient independent) [34] .

Cognitive Function Assessment

The Montreal Cognitive Assessment (MOCA) was administered as a short screening instrument to evaluate participants’ cognitive function. It has a score ranging from 0 to 30; a score of less than 26 is suggestive of cognitive impairment [9].

Statistical analysis

Data are reported as medians and interquartile ranges (IQR), means and standard deviations (SD), or counts and percentages (%) as appropriate. Comparisons between groups were made using t-tests, Mann-Whitney tests, Chi-square, or Fisher exact tests, dictated by data type and distribution. Pearson or Spearman correlations were used to test correlations between continuous variables. P values < 0.05 were considered significant for all statistical analyses in this study. All analyses were performed using the Statistical Package of Social Sciences (SPSS) version 21 for Windows (IBM Corp., Armonk, NY, USA).

## Results

Patient selection and characteristics

241 out of 447 patients receiving dialysis during the study period were successfully recruited into the study: 164 treated with high flux HD and 77 treated with HDF. Overall, among study patients, 66% were male, 62.7% were married, 43.2% were with less than high school, and 72.6% with arterio-venous fistula (AVF). Among those treated with high flux HD, 62.2% were male, 59.8% were married, 43.3% were with less than high school, and 70.1% with AVF. Among those treated with HDF, these percentages were 74%, 68.8%, 42.86%, and 77.9%, respectively. There was no statistically significant difference between both groups regarding demographic data except occupational status (Table 1).

**Table 1:** Comparison of the Demographic and Clinical Data of the studied groups of patients Non parametric data were expressed by median(range), parametric data expressed by;  mean±SD. Association between categorical variables was tested using Chi-square test while Fischer exact test was used when expected cell count was less than 5. Independent T sample test and Mann-whitney test were used when appropriate.; HD – hemodialysis, HDF – hemodiafiltration, p – p-vaue, n(%): number(percentage), BMI - body mass index, SAR - Saudi riyal, AVF - arteriovenous fistula, AVG - arteriovenous graft, HD – hemodialysis, HDF -hemodiafiltration. *- statictically significant a - comparison between HD vs. HDF groups

Parameters	Overall	HD group	HDF group	p^a^
Group size (n)	241	164	77	
Age/years median(min-max)	47(18-85)	48(18-80)	46(19-85)	0.83
Gender				
Male; n(%)	159(66)	102(62.2)	57(74)	0.08
Female; n(%)	82(34)	62(37.8)	20(26)	
Marital Status				
Single; n(%)	58(24.1)	43(26.2)	15(19.5)	0.49
Married; n(%)	151(62.7)	98(59.8)	53(68.8)	
Widow; n(%)	18(7.5)	14(8.5)	4(5.2)	
Divorced; n(%)	14(5.8)	9(5.5)	5(6.5)	
Educational Level				
Less than High School; n(%)	104(43.2)	71(43.3)	33(42.86)	0.81
High School; n(%)	58(24.1)	39(23.8)	19(24.68)	
Bachelor Degree; n(%)	65(27)	46(28)	19(24.68)	
Post Bachelor; n(%)	14(5.8)	8(4.9)	6(7.79)	
Occupation				
Retired; n(%)	67(27.8)	44(26.8)	23(29.9)	0.04*
Unemployed; n(%)	80(33.2)	63(38.4)	17(22.1)	
Housewife; n(%)	20(8.3)	11(6.7)	9(11.7)	
Student; n(%)	7(2.9)	6(3.7)	1(1.3)	
Administration; n(%)	23(9.5)	13(7.9)	10(13)	
Professional; n(%)	29(12)	15(9.1)	14(18.2)	
Others	15(6.2)	12(7.3)	3(3.9)	
Income SAR/month				
<5000; n(%)	120(49.8)	85(51.8)	35(45.5)	0.64
5000-10000; n(%)	76(31.5)	49(29.9)	27(35.1)	
10000-20000; n(%)	37(15.4)	37(15.4)	14(18.2)	
>20000; n(%)	7(2.9)	6(3.7)	1(1.3)	
Refused; n(%)	1(0.4)	1(0.6)	1(0.00)	
Dialysis Access				
AVF; n(%)	175(72.6)	115(70.1)	60(77.9)	0.12
AVG; n(%)	12(5)	7(4.3)	5(6.5)	
Jugular; n(%)	43(17.84)	32(19.5)	11(14.3)	
Subclavian; n(%)	9(3.73)	9(5.5)	0(0.00)	
Femoral; n(%)	2(0.83)	1(0.6)	1(1.3)	
Dialysis Duration/month median(min-max)	36(1-348)	36(1-348)	60(1-264)	0.07

Clinical examination, associated diseases and Charlson comorbidity index

Overall, among study patients, mean BMI was 26.64±6.7, 30.3% had diabetes, 84.2% had hypertension, 18.3% had coronary artery disease, and 17.8% had peripheral vascular disease. Among those treated with high flux HD, BMI was 25.8±6.5, 31.1% had diabetes, 82.3% had hypertension, 17.7% had coronary artery disease, and 13.3% had peripheral vascular disease. Among those treated with HDF, these values were 28.5±6.8, 28.6%, 88.3%, 19.5%, and 16.9% respectively. The HDF patients were larger weight, taller and with higher BMI (p=0.049, 0.001, 0.003, respectively). There was no significant difference between the two groups regarding other associated clinical disorders with overall median Charlson comorbidity index 4 (2-10) (Table 2).

**Table 2:** Comparison of the Associated diseases, comorbidities and Charlson comorbidity index of the studied groups of patients Non parametric data were expressed by median(range). Association between categorical variables was tested using Chi-square test while Fischer exact test was used when expected cell count was less than 5. Independent T sample test and Mann-whitney test were used when appropriate.; HD – hemodialysis, HDF – hemodiafiltration, n(%) - number(percentage), SD – standard deviation,  DM - diabetes mellitus, HTN – hypertension, HCV -hepatitis C virus, TTT: treatment, HBV - hepatitis B virus, *- statictically significant a - comparison between HD vs. HDF groups

Parameters	Overall	HD group	HDF group	p^a^
Number of patient (n)	241	164	77	
Height/meter; mean±SD	1.64±0.1	1.63±0.1	1.66±0.1	0.05*
Weight/kg; mean±SD	71.4±20.7	68.2±20.5	77.8±19.9	<0.01*
BMI kg/m2; mean±SD	26.64±6.7	25.8±6.5	28.5±6.8	<0.01*
DM; n(%)	73(30.3)	51(31.1)	22(28.6)	0.69
DM Duration; year Median (min-max)	10(2-30)	7.5(2-20)	14(230)	0.226
HTN; n(%)	203(84.2)	135(82.3)	68(88.3)	0.23
HTN Duration; year Median (min-max)	6.5(1-20)	6.5(1-20)	6(1-15)	0.381
HCV; n(%)	12(5)	8(4.9)	4(5.2)	1.00
HCV TTT; n(%)	8(3.3)	4(2.4)	4(5.2)	0.27
HBV; n(%)	9(3.7)	5(3)	4(5.2)	0.47
Coronary artery disease; n(%)	44(18.3)	29(17.7)	15(19.5)	0.72
Congested heart failure; n(%)	14(5.8)	11(6.8)	3(3.9)	0.56
Chronic liver disease; n(%)	16(6.6)	8(4.9)	8(10.4)	0.16
Chronic obstructive pulmonary disease; n(%)	7(2.9)	6(3.7)	1(1.3)	0.44
Psychological disorders; n(%)	9(3.7)	7(4.3)	2(2.6)	0.72
Depression; n(%)	11(4.6)	8(4.9)	3(3.9)	1.00
Cerebrovascular accident; n(%)	19(7.9)	14(8.5)	5(6.5)	0.80
Transient ischemic attack; n(%)	11(4.6)	2(1.2)	0(0.00)	1.00
Malignancy; n(%)	2(0.8)	5(3)	2(2.6)	1.00
Peripheral vascular diseases; n(%)	43(17.8)	30(13.3)	13(16.9)	0.86
Peptic ulcer; n(%)	16(6.6)	13(7.9)	3(3.9)	0.28
Connective tissue diseases; n(%)	22(9.1)	18(11)	4(5.2)	0.23
Skin ulcers; n(%)	2(0.8)	1(0.6)	1(1.3)	0.59
Charlson comorbidity index median(min-max)	4(2-10)	4(2-10)	4(2-9)	0.66

Biochemical and laboratory parameters

There was no significant difference in the incidence of anaemia, mineral bone disease and dialysis adequacy outcomes between the two groups (Table 3).

**Table 3:** Laboratory characteristic All parameters were expressed as median (minimum-maximum) unless stated otherwise. HD - hemodialysis, HDF – hemodiafiltration, p – p-value, n – number, Hb – haemoglobin, gm – gram, dl – deciliter, ug – nanogram, L – litre, mg- milligram, PTH – parathormone, IU – international units, HDL – high-density lipoprotein, LDL – low-density lipoprotein, BUN – blood urea nitrogen, Kt/V – K is dialyzer clearance of urea, t – time, V – the volume of distribution of urea, approximately equal to patient’s total body water, AST – aspartate transaminase, ALT – alanine transaminase *- statictically significant a - comparison between HD vs. HDF groups

Parameters	Overall	HD group	HDF group	p^a^
Group size (n)	241	164	77	
Anemia Parameters				
Hb (gm/dl) median(min-max)	11(5-14.7)	11(5.6-14.4)	11.1(5-14.7)	0.63
Hematocrit (%)median(min-max)	33.9(10-45)	33.7(10.2-45.4)	34.4(10.9-45.4)	0.42
Ferritin (ug/L) median(min-max)	452(11.98-2348)	455.45(15-2348)	412.3(11.98-1971)	0.08
TSAT (%) median(min-max)	32(9-84)	31(11-84)	32(9-58)	0.63
Bone minerals parameters				
Calcium (mg/dl) median(min-max)	9(6.4-12.5)	9(6.4-12.5)	8.9(7.8-10.5)	0.82
Phosphate (mg/dl) median(min-max)	5.1(2.53-11)	5.1(2.78-10.6)	5.17(2.53-11)	0.77
Vitamin D (ng/ml) median(min-max)	16.1(4-89.26)	15.9(5.15-89.26)	16.75(4-88.4)	0.61
PTH (pg/ml) median(min-max)	530(0.71-2500)	502.4(7.24-2500)	631.45(0.71-2500)	0.11
Alkaline phosphatase (IU/L) median(min-max)	114(39-1000)	114(39-985)	114.5(40-1000)	0.07
Lipid profile				
Cholesterol (mg/dl) median(min-max)	196(147-391)	189(147-391)	199(159-274)	0.26
HDL (mg/dl) median(min-max)	41(22-69)	41(23-69)	41(22-63)	0.66
LDL (mg/dl) median(min-max)	134(63-218)	127(63+218)	141(68-191)	0.09
Triglycerides (mg/dl) median(min-max)	187(133-650)	186.5(133-422)	193(137-650)	0.95
Adequacy parameters				
Creatinine (mg/dl); mean±SD	10.1±2.9	10.2±3.03	9.94±2.81	0.59
Pre BUN (mg/dl) median(min-max)	57(13-124)	57(14.7-124)	57(13-93)	0.69
Post BUN (mg/dl) median(min-max)	15(3.2-69)	15(3.2-69)	14(3.9-38.2)	0.23
Kt/V median(min-max)	1.56(0.64-2.83)	1.54(0.64-2.83)	1.63(.08-2.74)	0.1
Liver Function				
AST (IU) median(min-max)	13(5-68)	13(5-68)	13(5-46)	0.31
ALT (IU) median(min-max)	12(5-126)	12(5-126)	10(5-42)	0.09
Albumin (gm/dl) median(min-max)	4.1(1.7-5)	4.1(1.7-5)	4.1(2.9-5)	0.53

Activities of daily living

Katz index showed that 85.9% of patients were functionally independent, 87.2% of those on HD versus 83.1% of those on HDF. Moreover, the overall result of the IADL score showed that about 57% of patients were high-functioning and independent (66.7% males vs 37.8% females). Among those treated with high flux HD, 59% were high-functioning and independent (57.8% males vs 40.3% females). Among those on HDF, 68.8% were high-functioning and independent (82.5% males vs 30% females). There was no significant difference regarding daily living activity assessed either by Katz score or IADL score (p =0.711, 0.1, respectively) (Table 4).

**Table 4:** Comparison of Basic daily activity score, IADL score and MOCA score the studied group of patients Non-parametric data were expressed by median(minimum-maximum). Association between categorical variables was tested using Chi-square test while Fischer exact test was used when expected cell count was less than 5. acomparison between HD Vs. HDF groups b No significant difference as regard IADL score based on gender either for those in HD or HDF HD – hemodialysis, HDF – hemodiafiltration, p – p-value, HD – hemodialysis, HDF – hemodiafiltration, n – number, min – minimum, max – maximum, IADL - Instrumental Activities of Daily Living Scale. MOCA - Montreal Cognitive Assessment. *- statictically significant

Parameter	Overall		HD group		HDF group		p^a^
Group size (n)	241		164		77		
Basic daily activity score							
Median(min-max)	6(0-6)		6(0-6)		6(1-6)		0.52
score 0; n(%)	1(0.4)		1(0.6)		0(0.00)		0.71
score 1; n(%)	5(2.1)		3(1.8)		2(2.6)		
score 2; n(%)	6(2.5)		6(3.7)		0(0.00)		
score 3; n(%)	4(1.7)		3(1.8)		1(1.3)		
score 4; n(%)	3(1.2)		1(0.6)		2(2.6)		
score 5; n(%)	15(6.2)		7(4.3)		8(10.4)		
score 6; n(%)	207(85.9)		143(87.2)		64(83.1)		
IADL score							
Gender	Male	Female	Male	Female	Male	Female	
Number(%)	159(66%)	82(34%)	102(62.2%)	62(37.8%)	57(74%)	20(26%)	
Median(min-max)	5(0-5)	5.5(0-8)	5(0-5)	5(0-8)	5(1-5)	6(0-8)	<0.01*^b^
score 0; n(%)	1(0.6)	3(3.7)	1(1)	2(3.2)	0	1(5)	0.1
score 1; n(%)	5(3.1)	3(3.7)	3(2.9)	2(3.2)	2(3.5)	1(5)	
score 2; n(%)	6(3.8)	5(6.1)	5(4.9)	5(8.1)	1(1.8)	0	
score 3; n(%)	15(9.4)	5(6.1)	11(10.8)	4(6.5)	4(7)	1(5)	
score 4; n(%)	26(16.4)	13(15.9)	23(22.5)	12(19.4)	3(5.3)	1(5)	
score 5; n(%)	106(66.7)	12(14.6)	59(57.8)	8(12.9)	47(82.5)	4(20)	
score 6; n(%)		6(7.3)		2(3.2)		4(20)	
score 7; n(%)		4(4.9)		2(3.2)		2(10)	
score 8; n(%)		31(37.8)		25(40.3)		6(30)	
MOCA score							
Median(min-max)	23(10-30)		23(10-30)		23(10-30)		0.66
score ≥26; n(%)	44(18.3)		31(18.9)		13(16.9)		1
score 18-25; n (%)	151(62.7)		101(61.6)		50(64.9)		
score 11-17; n(%)	43(17.8)		30(18.3)		13(16.9)		
score 6-10; n(%)	3(1.2)		2(1.2)		1(1.3)		

The study showed a significant negative correlation between MOCA score, age, Charlson comorbidity index, and low density lipoprotein (LDL) (p=0.0001, 0.0001, and 0.049, respectively). Moreover, there was a significant positive correlation between MOCA score, haemoglobin, education level, income and vitamin D levels (p=0.014, 0.032, 0.001, and 0.004) respectively (Table 5).

**Table 5:** Correlation between MOCA score, clinical and laboratory data of the studied group of patients HD – hemodialysis, HDF – hemodiafiltration, p – p-value, MOCA – Montreal Cognitive Assessment score,  BMI – body mass index, IADL - IADL – Instrumental Activities of Daily Living, Hb – haemoglobin, dl – deciliter, ng – nanogram, ml- millilitre, TSAT – transferrin saturation, mg – milligram, HDL – high-density lipoprotein, LDL – low-density lipoprotein, pg – picogram, BUN – blood urea nitrogen, Kt/V – K is dialyzer clearance of urea, t – time, V – the volume of distribution of urea, approximately equal to patient’s total body water,  AST – aspartate transaminase, ALT – alanine transaminase, u-units, l – litre, iu – international units *- statistically significant

	MOCA score	
Variable	Pearson Correlation	P
Clinical predictors		
Age (years)	-0.417	<0.01*
BMI	-0.002	0.98
Dialysis Duration/months	0.011	0.87
Charlson Comorbidity Index	-0.246	<0.01*
Others		
Education	0.138	0.32
Income	0.201	0.01
Activity Scores		
Daily Activity Score	-0.012	0.85
IADL Score	0.001	0.56
Laboratory Data		
Hb (gm/dl)	0.158	0.01*
Hematocrit (%)	-0.019	0.77
Ferritin (ng/ml)	-0.06	0.35
TSAT (%)	0.045	0.48
Cholestrol (mg/dl)	-0.055	0.43
HDL (mg/dl)	-0.021	0.76
LDL (mg/dl)	-0.137	0.05*
Triglyceride (mg/dl)	0.073	0.29
Calcium (mg/dl)	-0.090	0.16
Phosphate (mg/dl)	-0.024	0.71
Vitamin D (ng/ml)	0.187	<0.01*
Parathormone hormone (pg/ml)	-0.107	0.01*
Creatinine (mg/dl)	-0.067	0.30
BUN pre-dialysis (mg/dl)	-0.017	0.79
BUN post-dialysis (mg/dl)	0.052	0.42
Kt/V	0.008	0.91
AST (u/l)	0.037	0.57
ALT (u/l)	0.043	0.51
Albumin (gm/dl)	-0.063	0.33
Alkaline phosphatase (iu/l)	0.083	0.21

Linear regression analysis showed that age was the most independent predictor for cognitive function (p=0.0001) (Table 6).

**Table 6:** Regression analysis for the risk factors of cognitive impairment MOCA – Montreal Cognitive Assessment score, std error – standard error, LDL – low-density lipoprotein, VIT D – vitamin D, HB – haemoglobin, IADL – Instrumental Activities of Daily Living, *- statistically significant

Coefficients for the dependent variable (MOCA score)						
Model		Unstandardized Coefficients		Standardized Coefficients	t	Sig.
		B	Std. Error	Beta		
1	(Constant)	23.641	2.673		8.846	0.00
	Age	-.109-	.024	-.333-	-4.59-	0.00
	Charlson score	-.176-	.175	-.074-	-1.01-	0.31
	LDL	-.017-	.009	-.120-	-1.83-	0.07
	VIT D	.058	.025	.155	2.371	0.02*
	HB	.388	.193	.132	2.007	0.05*
	IADL score	.243	.174	.090	1.398	0.16

## Discussion

In this cross-sectional study, we evaluated the prevalence of cognitive function impairment among ESRD patients depending on different hemodialysis modalities for the first time in Saudi Arabia. 

In our study, patients on online HDF were larger in size with no significant differences between them and those on high flux hemodialysis regarding anaemia, bone mineral, dialysis adequacy parameters, lipid profile or LFT. The concept of HDF which combined both diffusive and convective transport in one dialysis modality have been known since 1970 [23]. Since then, the superiority of HDF versus HD is still debatable and has been discussed in many clinical studies [35-39].

Our results were similar to the previous studies, including the ESHOL, CONTRAST, and Turkish studies [35-38], which found no significant differences between HDF and HD patients in haemoglobin levels and dialysis adequacy. Furthermore, ESHOL, Turkish, and Locatelli et al. [35,38,39] found no superiority for HDF versus high flux HD regarding phosphate level. Movilli et al. found no significant differences between the two modalities as to calcium level [40]. However, in contrast to our results, previous studies showed improvement in hemoglobin, phosphate, and Kt/V in HDF patients compared to HD patients [25,41-43]. The discrepancies between our current results, our earlier results and literature may be attributed to the difference in the study design and the selection criteria.

Our study showed no significant differences between HDF patients and HD patients regarding daily activity. On the other hand, there was significantly higher IADL score among those on HDF. It is well known that patients on dialysis have limited physical activity which also decreases over time. A limited number of studies discuss the correlation between hemodialysis modality and dialysis patients’ daily activity. Nevertheless, Pecoits-Filho et al. concluded in their randomized control trial that there is no significant difference between HDF and HD regarding physical activities, despite the improvement in other parameters [44]. 

Previous studies demonstrated increasing frequency of cognitive functions impairment and deterioration of cognition in hemodialysis patients [8,45]. On the other hand, there is evidence that HD itself accelerates cognitive impairment compared to peritoneal dialysis [37-41].

Based on the MOCA score interpretation, the prevalence of cognitive functions impairment among our studied group of patients was 81.7% ranging from mild to severe cognitive impairment. These results were similar to Wolfgram et al. results, which found that 82.5% of his participants had CI, and 66% of them showed moderate to severe impairment [46]; whilst our study showed only 19% incidence of moderate to severe CI. This difference may be attributed to the difference in patients selection and the sample size. Wolfgram et al. recruited those aged >50 years, and the number of patients included in this study was 40 patients. Similarly to the prevalence of mild CI among our patients (62.7%), Pei et al. reported that 60.9% of hemodialysis patients had mild CI [47].

Regarding the effect of HDF versus high flux HD on cognitive dysfunction, we did not find any significant differences between the two modalities and to the best of our knowledge, there are no available published studies that discussed such a point.

As hemodialysis patients are more vulnerable to CI than others [3], identifying the risk factors of CI may help us implement strategies aiming to delay such illness progression and improve our patients’ quality of life. The older the age, the higher the Charlson comorbidity index, the lower the haemoglobin level, the higher the LDL level and the lower the vitamin D level, the worse the cognitive functions of our patients. Age, vitamin D level and Hb were the most independent predictors of cognitive functions impairment among HD patients.

Drew et al. concluded that older age was the only risk factor for cognitive functions impairment in HD patients [3]. Age, education level, history of stroke and hypertension, dialysis vintage, and single-pool Kt/V were the main risk factors of CI in HD patients, according to Luo et al. [48], while age and serum concentrations of Hb, cholesterol, and PTH were the main risk factors of CI as reported by Fadili et al. [49]. On the other hand, Shaffi et al. and Liu et al. concluded that low vitamin D level is associated with worse cognitive function impairment in HD patients [50,51].

This study has its limitations; the non-randomized, non-blinding confounding design, may have influenced the results. The number of HDF patients was ower than high flux HD patients. A large prospective double-blinded randomized control study is required to assess the magnitude of this problem and evaluate the possible correctable causes that could delay the disease’s progression.

## Conclusions

Cognitive function impairment is a common problem in hemodialysis with no superiority of online HDF versus high flux HD in improving cognitive functions. Cognitive functions impairment in hemodialysis is associated with multiple risk factors and treatment of anaemia, vitamin D deficiency and high LDL may help improve and delay the progression of CI in hemodialysis patients.

## References

[1] Gupta R (2004). Trends in hypertension epidemiology in India. J Hum Hypertens.

[2] Sehgal AR, Grey SF, DeOreo PB, Whitehouse PJ (1997). Prevalence, recognition, and implications of mental impairment among hemodialysis patients.

[3] Drew DA, Weiner DE, Tighiouart H, Duncan S, Gupta A, Scott T, Sarnak MJ (2017). Cognitive decline and its risk factors in prevalent hemodialysis patients. Am J Kidney Dis.

[4] Gupta A, Montgomery RN, Bedros V (2019). Subclinical cognitive impairment and listing for kidney transplantation. Clin J Am Soc Nephrol.

[5] Kurella M, Chertow GM, Luan J, Yaffe K (2004). Cognitive impairment in chronic kidney disease. J Am Geriatr Soc.

[6] Yaffe K, Ackerson L, Kurella Tamura M (2010). Chronic kidney disease and cognitive function in older adults: findings from the chronic renal insufficiency cohort cognitive study. J Am Geriatr Soc.

[7] Kurella Tamura M, Yaffe K (2011). Dementia and cognitive impairment in ESRD: diagnostic and therapeutic strategies. Kidney Int.

[8] Murray AM (2008). Cognitive impairment in the aging dialysis and chronic kidney disease populations: an occult burden. Adv Chronic Kidney Dis.

[9] Nasreddine ZS, Phillips NA, Bédirian V (2005). The Montreal Cognitive Assessment, MoCA: a brief screening tool for mild cognitive impairment. J Am Geriatr Soc.

[10] Patel M, Dasgupta I, Tadros G, Baharani J (2016). Cognitive impairment in hemodialysis patients: what can slow this decline?. Hong Kong J Nephrol.

[11] Farrall AJ, Wardlaw JM (2009). Blood-brain barrier: ageing and microvascular disease--systematic review and meta-analysis. Neurobiol Aging.

[12] Toyoda K, Fujii K, Fujimi S, Kumai Y, Tsuchimochi H, Ibayashi S, Iida M (2005). Stroke in patients on maintenance hemodialysis: a 22-year single-center study. Am J Kidney Dis.

[13] Casserly I, Topol EJ (2004). Convergence of atherosclerosis and Alzheimer's disease: inflammation, cholesterol, and misfolded proteins. Lancet.

[14] Breteler MM, van Amerongen NM, van Swieten JC (1994). Cognitive correlates of ventricular enlargement and cerebral white matter lesions on magnetic resonance imaging. The Rotterdam Study. Stroke.

[15] Arieff AI (1985). Aluminum and the pathogenesis of dialysis encephalopathy. Am J Kidney Dis.

[16] Scaini G, Ferreira GK, Streck EL (2010). Mechanisms underlying uremic encephalopathy. Rev Bras Ter Intensiva.

[17] Chen CY, Hung SY, Lee YJ, Lin YC, Pai CC (2016). Delayed onset of posterior reversible encephalopathy syndrome in a case of scleroderma renal crisis with maintenance hemodialysis: case report and literature review. Medicine (Baltimore).

[18] Brodaty H, Pond D, Kemp NM, Luscombe G, Harding L, Berman K, Huppert FA (2002). The GPCOG: a new screening test for dementia designed for general practice. J Am Geriatr Soc.

[19] Pickett JL, Theberge DC, Brown WS, Schweitzer SU, Nissenson AR (1999). Normalizing hematocrit in dialysis patients improves brain function. Am J Kidney Dis.

[20] Jassal SV, Devins GM, Chan CT, Bozanovic R, Rourke S (2006). Improvements in cognition in patients converting from thrice weekly hemodialysis to nocturnal hemodialysis: a longitudinal pilot study. Kidney Int.

[21] Sharp J, Wild MR, Gumley AI, Deighan CJ (2005). A cognitive behavioral group approach to enhance adherence to hemodialysis fluid restrictions: a randomized controlled trial. Am J Kidney Dis.

[22] Harciarek M, Biedunkiewicz B, Lichodziejewska-Niemierko M, Dębska-Ślizień A, Rutkowski B (2011). Continuous cognitive improvement 1 year following successful kidney transplant. Kidney Int.

[23] Henderson LW, Colton CK, Ford CA (1975). Kinetics of hemodiafiltration. II. Clinical characterization of a new blood cleansing modality. Transl Res.

[24] Nistor I, Palmer SC, Craig JC, Saglimbene V, Vecchio M, Covic A, Strippoli GF (2015). Haemodiafiltration, haemofiltration and haemodialysis for end-stage kidney disease. Cochrane Database Syst Rev.

[25] Abdelsalam M, Demerdash TM, Assem M (2021). Improvement of clinical outcomes in dialysis: no convincing superiority in dialysis efficacy using hemodiafiltration vs high-flux hemodialysis. Ther Apher Dial.

[26] Li PK, Cheng YL, Leung CB (2010). Effect of membrane permeability on inflammation and arterial stiffness: a randomized trial. Clin J Am Soc Nephrol.

[27] Jia P, Jin W, Teng J (2016). Acute effects of hemodiafiltration versus conventional hemodialysis on endothelial function and inflammation: a randomized crossover study. Medicine (Baltimore).

[28] Potier J, Bowry S, Canaud B (2017). Clinical performance assessment of CorDiax filters in hemodialysis and hemodiafiltration. Contrib Nephrol.

[29] Morena M, Jaussent A, Chalabi L (2017). Treatment tolerance and patient-reported outcomes favor online hemodiafiltration compared to high-flux hemodialysis in the elderly. Kidney Int.

[30] Karkar A, Abdelrahman M, Locatelli F (2015). A randomized trial on health-related patient satisfaction level with high-efficiency online hemodiafiltration versus high-flux dialysis. Blood Purif.

[31] Suwabe T, Barrera-Flores FJ, Rodriguez-Gutierrez R, Ubara Y, Takaichi K (2018). Effect of online hemodiafiltration compared with hemodialysis on quality of life in patients with ESRD: a systematic review and meta-analysis of randomized trials. PLoS One.

[32] von Elm E, Altman DG, Egger M, Pocock SJ, Gøtzsche PC, Vandenbroucke JP (2007). The Strengthening the Reporting of Observational Studies in Epidemiology (STROBE) statement: guidelines for reporting observational studies. Bull World Health Organ.

[33] Lawton MP, Brody EM (1969). Assessment of older people: self-maintaining and instrumental activities of daily living. Nurs Res.

[34] Katz S, Downs TD, Cash HR, Grotz RC (1970). Progress in development of the index of ADL. Gerontologist.

[35] Maduell F, Moreso F, Pons M (2013). High-efficiency postdilution online hemodiafiltration reduces all-cause mortality in hemodialysis patients. J Am Soc Nephrol.

[36] Grooteman MP, van den Dorpel MA, Bots ML (2012). Effect of online hemodiafiltration on all-cause mortality and cardiovascular outcomes. J Am Soc Nephrol.

[37] Penne EL, van der Weerd NC, van den Dorpel MA (2010). Short-term effects of online hemodiafiltration on phosphate control: a result from the randomized controlled Convective Transport Study (CONTRAST). Am J Kidney Dis.

[38] Ok E, Asci G, Toz H (2013). Mortality and cardiovascular events in online haemodiafiltration (OL-HDF) compared with high-flux dialysis: results from the Turkish OL-HDF Study. Nephrol Dial Transplant.

[39] Locatelli F, Altieri P, Andrulli S (2014). Phosphate levels in patients treated with low-flux haemodialysis, pre-dilution haemofiltration and haemodiafiltration: post hoc analysis of a multicentre, randomized and controlled trial. Nephrol Dial Transplant.

[40] Movilli E, Camerini C, Gaggia P (2011). Effect of post-dilutional on-line haemodiafiltration on serum calcium, phosphate and parathyroid hormone concentrations in uraemic patients. Nephrol Dial Transplant.

[41] Orasan RA, Patiu IM, Anghel D (2013). Variation of clinical and laboratory features in chronic dialysis patients treated with high-flux hemodialysis after switching to online hemodiafiltration. Int Urol Nephrol.

[42] Bonforte G, Grillo P, Zerbi S, Surian M (2002). Improvement of anemia in hemodialysis patients treated by hemodiafiltration with high-volume on-line-prepared substitution fluid. Blood Purif.

[43] Djuric PS, Jankovic A, Popovic J (2016). Survival benefit of hemodiafiltration compared with prolonged high-flux hemodialysis. Iran J Kidney Dis.

[44] Pecoits-Filho R, Larkin J, Poli-de-Figueiredo CE (2021). Effect of hemodiafiltration on measured physical activity: primary results of the HDFIT randomized controlled trial. Nephrol Dial Transplant.

[45] Gela YY, Getu AA, Adane A (2021). Cognitive impairment and associated factors among chronic kidney disease patients: a comparative cross-sectional study. Neuropsychiatr Dis Treat.

[46] Wolfgram DF, Sunio L, Vogt E, Smith HM, Visotcky A, Laud P, Whittle J (2014). Haemodynamics during dialysis and cognitive performance. Nephrology (Carlton).

[47] Pei X, Lai S, He X (2019). Mild cognitive impairment in maintenance hemodialysis patients: a cross-sectional survey and cohort study. Clin Interv Aging.

[48] Luo Y, Murray AM, Guo YD (2020). Cognitive impairment and associated risk factors in older adult hemodialysis patients: a cross-sectional survey. Sci Rep.

[49] Fadili W, Al Adlouni A, Louhab N, Habib Allah M, Kissani N, Laouad I (2014). Prevalence and risk factors of cognitive dysfunction in chronic hemodialysis patients. Aging Ment Health.

[50] Shaffi K, Tighiouart H, Scott T, Lou K, Drew D, Weiner D, Sarnak M (2013). Low 25-hydroxyvitamin D levels and cognitive impairment in hemodialysis patients. Clin J Am Soc Nephrol.

[51] Liu GL, Pi HC, Hao L (2015). Vitamin D status is an independent risk factor for global cognitive impairment in peritoneal dialysis patients. PLoS One.

